# Nationwide Monitoring for *Plasmodium falciparum* Drug-Resistance Alleles to Chloroquine, Sulfadoxine, and Pyrimethamine, Haiti, 2016–2017

**DOI:** 10.3201/eid2605.190556

**Published:** 2020-05

**Authors:** Eric Rogier, Camelia Herman, Curtis S. Huber, Karen E.S. Hamre, Baby Pierre, Kimberly E. Mace, Jacquelin Présumé, Gina Mondélus, Ithamare Romilus, Tamara Elismé, Thomas P. Eisele, Thomas Druetz, Alexandre Existe, Jacques Boncy, Jean F. Lemoine, Venkatachalam Udhayakumar, Michelle A. Chang

**Affiliations:** Centers for Disease Control and Prevention, Atlanta, Georgia, USA (E. Rogier, C. Herman, C.S. Huber, K.E.S. Hamre, K.E. Mace, V. Udhayakumar, M.A. Chang);; Ministère de la Santé Publique et de la Population, Port-au-Prince, Haiti (B. Pierre, J. Présumé, G. Mondélus, I. Romilus, T. Elismé, A. Existe, J. Boncy, J.F. Lemoine);; Tulane University School of Public Health and Tropical Medicine, New Orleans, Louisiana, USA (T.P. Eisele);; University of Montreal School of Public Health, Montreal, Canada (T. Druetz)

**Keywords:** *Plasmodium falciparum*, Haiti, drug resistance, chloroquine, sulfadoxine, pyrimethamine, malaria, parasites

## Abstract

Haiti is striving for zero local malaria transmission by the year 2025. Chloroquine remains the first-line treatment, and sulfadoxine/pyrimethamine (SP) has been used for mass drug-administration pilot programs. In March 2016, nationwide molecular surveillance was initiated to assess molecular resistance signatures for chloroquine and SP. For 778 samples collected through December 2017, we used Sanger sequencing to investigate putative resistance markers to chloroquine (*Pfcrt* codons 72, 74, 75, and 76), sulfadoxine (*Pfdhps* codons 436, 437, 540, 581, 613), and pyrimethamine (*Pfdhfr* codons 50, 51, 59, 108, 164). No parasites harbored *Pfcrt* point mutations. Prevalence of the *Pfdhfr* S108N single mutation was 47%, and we found the triple mutant *Pfdhfr* haplotype (108N, 51I, and 59R) in a single isolate. We observed no *Pfdhps* variants except in 1 isolate (A437G mutation). These data confirm the lack of highly resistant chloroquine and SP alleles in Haiti and support the continued use of chloroquine and SP.

The island of Hispaniola remains the last location with endemic malaria in the Caribbean region, and ongoing elimination efforts aim to achieve zero cases from local transmission by the year 2025 (http://www.malariazeroalliance.org) (*1*). The western nation within Hispaniola, Haiti, has renewed interest in malaria elimination after the devastating 2010 earthquake in the southern part of the country, and local and international partners are collaborating to achieve this goal. In Haiti, chloroquine with a single dose of primaquine remains the first-line treatment for uncomplicated *Plasmodium falciparum* malaria, and strong evidence indicates that parasites in the country remain largely sensitive to chloroquine (*2*–*4*), although some researchers recommend monitoring patients after chloroquine treatment to ensure parasite clearance (*5*,*6*). Concerns about the potential importation of chloroquine-resistant *P. falciparum* haplotypes into Haiti persist because of the proximity to South America and because travelers from Africa commonly visit Haiti (*1*,*7*,*8*).

Until recently, sulfadoxine/pyrimethamine (SP) was the second-line treatment for malaria in Haiti. SP works to inhibit the protozoal folate pathway, can be administered inexpensively in a single dose, is generally well-tolerated by the recipient population (*9*,*10*), and has a long half-life in humans (both drugs remain in plasma well beyond 1 month) (*11*). These attributes have made SP an attractive option for malaria chemoprophylaxis (*9*,*12*) and other types of population-based mass drug administration (MDA) campaigns (*13*). Other antimalarial medications such as dihydroartemesinin/piperaquine are used in other settings for MDA but are hampered by the need for multiday dosing. As a nation moves toward malaria elimination and very low *P. falciparum* transmission rates, the residual infections in the human population are predominantly asymptomatic and even below the limit of detection for diagnostic tests (*14*,*15*). Having an effective antiparasitic drug therapy capable of clearing this malaria reservoir through MDA could be a tool to interrupt *P. falciparum* transmission in a population (*16*).

In areas of the world with low levels of *P. falciparum* transmission, in vivo drug efficacy studies with sufficient statistical power are difficult to perform; thus, screening parasite populations for well-characterized genetic drug-resistance markers can provide a viable alternative to monitor the emergence of drug-resistant haplotypes (*17*,*18*). In particular, the *Pfcrt* K76T polymorphism is known to be the strongest single molecular predictor of chloroquine resistance; numerous global studies have linked this mutation with clinical failure (*18*–*20*). For SP treatment, each antifolate compound has multiple putative molecular markers that confer resistant phenotypes; mutations in the *Pfdhps* gene show the highest risk for resistance to sulfadoxine, and *Pfdhfr* mutations show the highest risk for resistance to pyrimethamine (*21*). More important, beyond a single missense mutation, multiple concurrent mutations (>3) in a *P. falciparum* isolate’s *Pfdhps* and *Pfdhfr* genes have been shown to be a robust predictor of parasite resistance to SP (*18*).

As Haiti moves toward malaria elimination, verification of drug efficacy will be crucial for ensuring *Plasmodia* parasites can be successfully cleared from infected persons in the population. However, because malaria prevalence has declined to low levels in Haiti, conducting in vivo therapeutic efficacy studies becomes increasingly difficult. Therefore, routine molecular surveillance for markers of antimalarial drug resistance was established at the 11 sentinel sites in Haiti in 2016. In this article, we outline the molecular surveillance data related to chloroquine- and SP-resistant alleles from samples collected during the first 2 years of surveillance (2016–2017).

## Materials and Methods

### Surveillance Network and Population

We selected a health facility in each of Haiti’s 10 administrative departments, plus an 11th site in Grand Anse (the department that consistently has the highest number of reported malaria cases [*1*]), as sentinel sites to participate in antimalarial molecular resistance marker surveillance. Treatment-seeking persons of all ages who sought care at any of the 11 sentinel sites for symptoms of malaria and who also tested positive for malaria by either rapid diagnostic test (RDT) or microscopy were eligible to participate and provide a blood sample for malaria testing. Experienced microscopists performed thick-smear microscopy on Giemsa-stained slides. RDT testing was performed with RDTs available at each health facility and would have been by 1 of the 3 best-in-class HRP2-based tests: First Response Malaria Ag HRP2 (Premier Medical Corporation, http://premiermedcorp.com), CareStart Malaria HRP2 (Pf) (Access Bio, http://www.accessbio.net), and SD Bioline Malaria Ag Pf (Standard Diagnostics, https://sdinc.en). Patients who were clinically unstable and requiring urgent medical care were ineligible for participation. 

We collected 2,016 samples from consenting patients from the 11 sentinel sites during March 2016–December 2017. The protocol for molecular surveillance was approved by the Haitian Ministry of Public Health and Population Bioethics Committee as a nonresearch programmatic activity. This protocol was also reviewed by the US Centers for Disease Control and Prevention (CDC) Center for Global Health and approved as a nonresearch surveillance activity. Blood specimens were collected only when participants (parents or guardians for children) consented to participate.

Because the sentinel health facility in Artibonite (Centre de Santé Clinique Jolivert) had not provided any malaria-positive specimens during 2016–2017, we chose samples from a separate malaria prevalence survey conducted in southern Artibonite in April 2017 from 2 health facilities ≈1 km apart to represent genotypes from this important malaria transmission area. This survey in Artibonite was approved by Haiti’s Ministry of Public Health and Population Bioethics Committee and the institutional review boards of Tulane University and the London School of Hygiene and Tropical Medicine. Adult participants provided written consent, consent for children (<18 years) was provided by a parent or guardian, and children >6 years of age gave written assent to participate. Persons 16 or 17 years of age who were married, a head of household, or a parent were considered mature minors and consented directly. Thumbprint consent or assent (countersigned by a witness) was used for illiterate participants. Persons <6 months of age or who required immediate medical attention were excluded.

### Sample Collection and DNA Extraction in Laboratory

For consenting participants in sentinel surveillance and cross-sectional survey study sites, health facility workers drew a sample of ≈200 μL by fingerprick onto Whatman 903 Protein Saver cards (GE Healthcare Life Sciences, https://www.gelifesciences.com). To ensure consistency in blood sample collection, all health facility workers had previously been trained on appropriate dried blood spot sample preparation. Each filter paper was air-dried overnight and individually stored in a sealed plastic bag containing a desiccant packet. Filter papers were stored at health facilities at room temperature away from sunlight until transfer. Three times per year, sentinel site samples were transferred to Haiti’s National Public Health Laboratory (Laboratoire National de Santé Publique [LNSP]). For the separate Artibonite survey, samples were transported to LNSP on a weekly basis. 

At LNSP, filter paper cards were cut in half, with 1 section remaining at LNSP and 1 shipped to CDC (Atlanta, Georgia, USA) for molecular analysis and sequencing. On the Whatman 903 card, the center circle was not filled with blood to allow cutting of the card down the middle without introducing DNA contamination risk. Scissors were wiped with 70% ethanol between card cuttings. DNA extraction was performed by using the DNA Mini Kit (QIAGEN, https://www.qiagen.com) according to the manufacturer’s protocol, with the exception that double the amount of filter paper blood and proteinase K were used. After elution, DNA samples were kept at 4°C for short-term storage and −40°C for long-term storage.

### *P. falciparum* Photo-Induced Electron Transfer–PCR and Gene Sequencing

We confirmed the presence and quantity of *P. falciparum* DNA by using the *P. falciparum* photo-induced electron transfer PCR (PET-PCR) real-time assay as described previously (*22*). We multiplexed primer targets for *Plasmodium* genus and *P. falciparum* 18S rDNA in a single reaction, which we then amplified for 45 cycles. We determined cycle threshold (C_t_) on the basis of the beginning of the log phase increase in fluorescence intensity, and we considered a C_t_ value <40.0 to be DNA-positive signal. We converted C_t_ values to estimated parasite densities on the basis of a separate DNA dilution series of 3D7 and Dd2 *P. falciparum* blood cultures that had been dried on Whatman 903 filter paper and on the basis of DNA extracted according to the same protocol we have described. We estimated parasite density by extrapolating from C_t_ values on the basis of the average regression curve of the 2 *P. falciparum* culture strains (*22*).

We performed Sanger sequencing for *Pfcrt*, *Pfdhfr*, and *Pfdhps* as described previously (*2*) by using primer sets and reaction conditions for nested PCR and sequencing PCR (Appendix Table 1). We amplified the *Pfcrt* and *Pfdhfr* genes by using primary and secondary nested PCR reactions and amplified *Pfdhps* genes by using a single PCR reaction. We conducted all amplification reactions by using the Expand High Fidelity PCR buffer (Roche, https://www.roche.com) and the Sigma dNTP kit (Sigma Aldrich, https://www.sigmaaldrich.com), with addition of 1μL of each primer (at 15 mmol/L) and 2 μL DNA per reaction, yielding a final reaction volume of 20 μL. We subjected amplified products to a sequencing PCR with the Applied Biosystems Big Dye kit (ThermoFisher, https://www.thermofisher.com) with 1 μL of 15 mmol/L primer (Appendix Table 1). We cleaned sequencing PCR products by ethanol precipitation and resuspended them in Applied Biosystems Hi-Di formamide (*2*). We sequenced samples on an Applied Biosystems 3130 XL Genetic Analyzer and read gene sequences on MacVector 7.2 software (MacVector, https://macvector.com).

## Results

**Figure 1:**
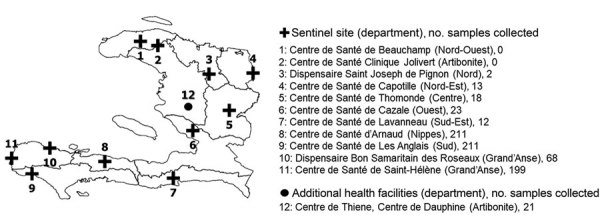
Healthcare facility locations and number of samples tested for malaria genetic drug-resistance surveillance, Haiti, 2016–2017. Eleven sentinel sites throughout Haiti were selected. Nine of these sites, plus additional healthcare facilities covered in a survey conducted in Artibonite Department, provided a total of 778 samples for molecular analysis.

For samples collected during March 2016–December 2017, 757 were analyzed for molecular drug resistance markers associated with chloroquine and SP resistance. Of 11 sentinel sites participating in the program, 9 provided samples (Figure 1). A total of 21 samples for Artibonite were gathered through the separate survey in April 2017. Most samples analyzed (689, 88.6%) came from four health facilities in the western half of the southern peninsula (departments: Sud, Grand Anse, and Nippes).

**Figure 2:**
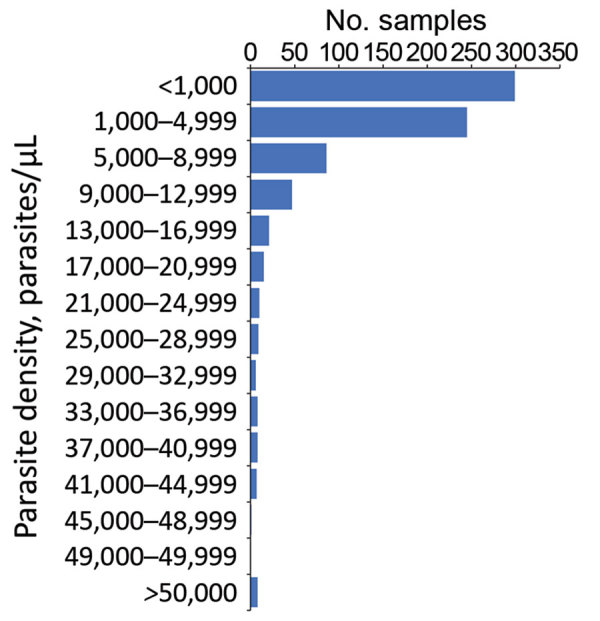
Frequency of estimated parasite densities for dried blood spot samples collected, Haiti, 2016–2017.

As most persons were enrolled through positivity to an antigen-based RDT, presence of malaria DNA with a person’s blood sample was confirmed through PCR reactions at both the LNSP and the CDC malaria laboratory. The 2 laboratories showed good concordance in detection of parasite DNA through quantitative PET-PCR assays (Appendix Figure 1). Quantitative estimates for parasite densities in the 778 samples analyzed found the majority of *P. falciparum* infections to be at low estimated parasite densities; 299 (38.4%) were estimated at a parasite density of <1,000 p/μL blood and 544 (69.9%) <5,000 p/μL blood (Figure 2). Only 8 (1.0%) of the samples analyzed were estimated to contain a parasite density of >50,000 p/μL blood.

Of 778 samples analyzed, we successfully sequenced 741 (95.2%) for reporting of *Pfcrt* polymorphisms at codons 72, 74, 75, and 76 (Table; Appendix Table 2). All samples successfully sequenced showed the CVMNK genotype (codons 72–76), indicating an absence of the molecular markers for chloroquine resistance in the *Pfcrt* gene. Sequencing for *Pfdhfr* was less successful; 548 (70.4%) of all samples analyzed provided results for codons 50, 51, 59, 108, and 164 (Appendix Table 2). We found No polymorphisms at the C50R and I164L codons. A single isolate from the Cazale facility in the Ouest department showed a triple mutation in the N51I, C59R, and S108N codons, but we observed no other N51I or C59R mutants in other isolates. Of interest, 46.9% (257/548) of *Pfdhfr* sequenced isolates carried the S108N mutation, associated with low level pyrimethamine resistance. The S108N mutation was found in all sites except the Pignon facility in the Nord department (although this facility only provided 2 samples) and ranged from 6% to 67% of isolates sequenced from each health facility (Table). Sequencing of *Pfdhps* was least successful, with 406 (52.2%) samples providing interpretable results. Not considering the 540 codon, we successfully 465 (59.8%) samples analyzed sequenced for *Pfdhps*. The only mutations in *Pfdhps* were for the five considered codons, except for a single polymorphism of A437G found in 1 sample from the Les Anglais facility in Sud department.

**Table:** Serum samples indicating putative malaria drug-resistance codon mutations for genes *Pfcrt*, *Pfdhfr*, and *Pfdhps*, by healthcare facility, Haiti, 2016–2017*

Site (no. samples)	*Pfcrt*†	*Pfdhfr*						*Pfdhps*								
		C50R	N51I	C59R	S108N	I164L		S436X‡		A437G		K540E	A581G		A613X§	
Arnaud (211)	ND	ND	ND	ND	132 (63)	ND		ND	ND		ND			ND		ND
Capotille (13)	ND	ND	ND	ND	1 (8)	ND		ND	ND		ND			ND		ND
Cazale (23)	ND	ND	1 (4)	1 (4)	13 (57)	ND		ND	ND		ND			ND		ND
Thienne/Dauphine (21)	ND	ND	ND	ND	14 (67)	ND		ND	ND		ND			ND		ND
Lavanneau (12)	ND	ND	ND	ND	1 (8)	ND		ND	ND		ND			ND		ND
Les Anglais (211)	ND	ND	ND	ND	27 (13)	ND		ND	1 (0.5)		ND			ND		ND
Pignon (2)	ND	ND	ND	ND	ND	ND		ND	ND		ND			ND		ND
Roseaux (68)	ND	ND	ND	ND	15 (22)	ND		ND	ND		ND			ND		ND
Sainte-Hélène (199)	ND	ND	ND	ND	53 (27)	ND		ND	ND		ND			ND		ND
Thomonde (18)	ND	ND	ND	ND	1 (6)	ND		ND	ND		ND			ND		ND
Total (778)	ND	ND	1 (0.2)	1 (0.2)	257 (47)	ND		ND	1 (0.2)		ND			ND		ND

*Values are no. (%) unless indicated. ND, not detected. †Mutations in any of the *Pfcrt* C72S, M74I, N75E, or K76T codons. ‡S436A, S436Y, or S436F mutations. §A613S or A613T mutations.

**Figure 3:**
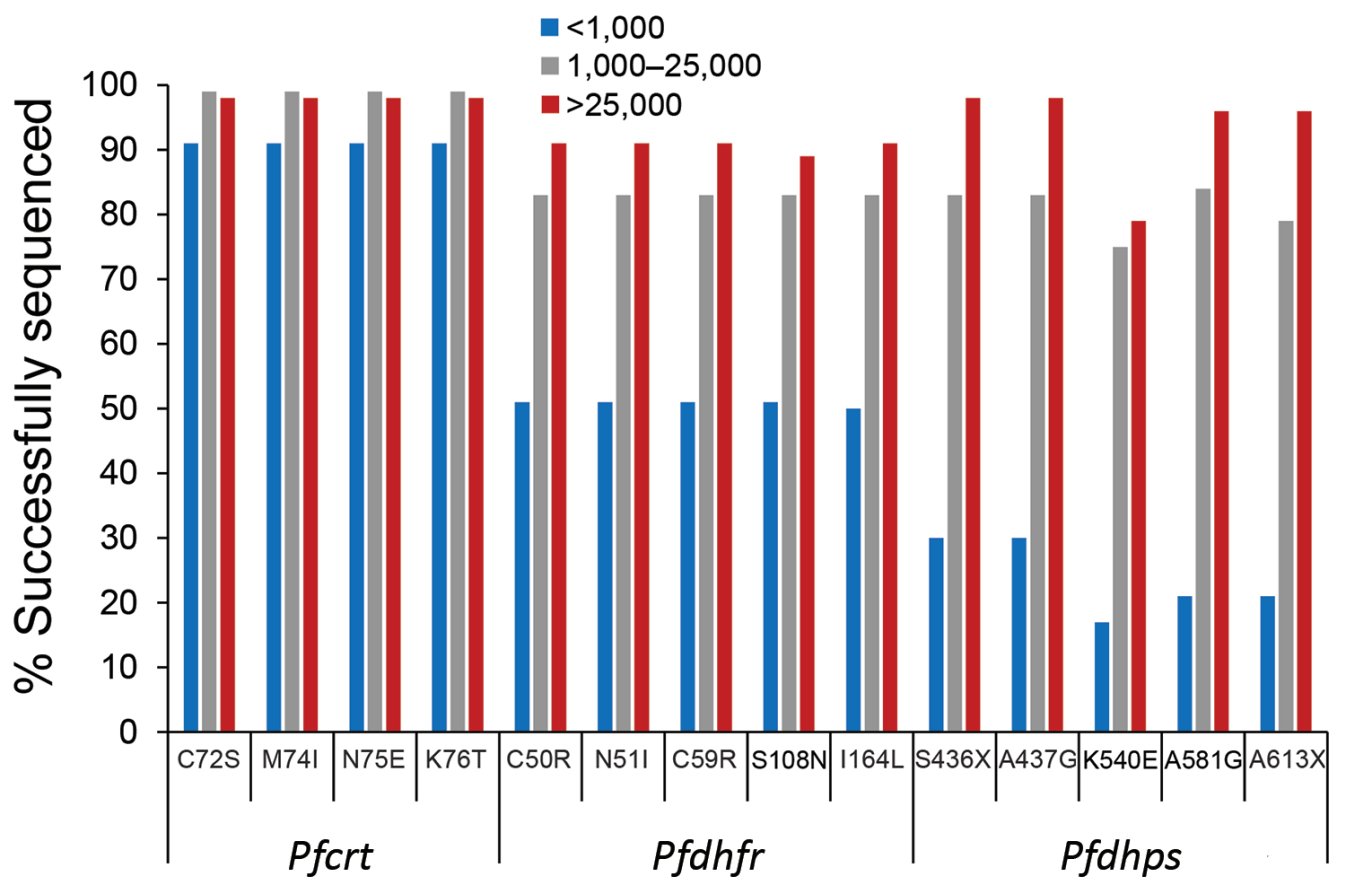
Success rate of reporting codon sequencing data by *Plasmodium falciparum* gene and estimated parasite density, Haiti, 2016–2017. Successful reporting rates are shown by estimated parasites densities of <1,000, 1,000–25,000, and >25,000 parasites/μL.

The rate of success for sequencing the *Pfcrt*, *Pfdhfr*, and *Pfdhps* genes was largely dependent upon the estimated parasite density via *P. falciparum* DNA content in the sample. At parasite densities >1,000 p/μL blood, all codons were reported for *Pfcrt* in >98% of samples (Figure 3). Even at estimated densities <1,000 p/μL blood, reportable results were possible for the *Pfcrt* codons in 91% of the isolates. More striking differences in success of gene sequencing were observed for *Pfdhfr* and *Pfdhps* genes. Patient samples with estimated densities >1,000 p/μL blood generated reportable results for the 10 codons (5 for *Pfdhfr* and 5 for *Pfdhps*) >75% of the time, and in samples >25,000 p/μL blood sequencing was successful 89% of the time (if not including *Pfdhps* K540E at 79%). However, at densities <1,000 p/μL blood, the 5 *Pfdhfr* codons were only sequenced in 50% of samples and the 5 *Pfdhps* codons in 17% of samples.

## Discussion

Our study aimed to describe Haiti’s nationwide sentinel site surveillance program for monitoring antimalarial drug resistance for samples collected during 2016–2017 (*1*). Because patient samples will continue to be collected from these participating health facilities during the country’s progress toward malaria elimination, changes in the parasite population (e.g., changes in molecular signatures related to drug resistance) should be quickly detected through this surveillance system. Epidemiologic findings from the first 2 years of the surveillance program will be reported elsewhere.

We used a total of 778 samples, representing 9 of the 10 departments in Haiti (no samples were taken in the Nord-Ouest department), for this initial report to establish a molecular baseline for polymorphisms in the *P. falciparum* genes *Pfcrt*, *Pfdhfr*, and *Pfdhps*. Being able to draw from samples in many different areas of the country enables a higher confidence of genetic representation in identifying any drug-resistant populations; however, isolated foci of drug-resistant haplotypes might exist in the country. Because the RDT used in this survey relies on detection of histidine-rich protein (HRP) 2 for primary diagnosis, any *P. falciparum* isolates lacking functional HRP2 and HRP3 production might have been overlooked through this surveillance strategy (*23*). However, this possibility is remote, given that no evidence to date suggests any deletion of the HRP2 gene in this population. 

All persons in this study were enrolled at health facilities, so the data do not represent the asymptomatic or non–treatment seeking reservoir in Haiti, and our enrollment procedures might also have missed smaller foci of inbred or clonal *P. falciparum* drug-resistant haplotypes in the country outside of the catchment areas of these health facilities (*24*,*25*). Most (689 [88.6%]) of the samples tested were from patients enrolled in 1 of 4 health facilities in the southwest corner of Haiti. This proportion is indicative of current *P. falciparum* transmission in Haiti, where higher case counts have been observed in the departments of Grand Anse, Sud, and Nippes compared with the rest of the country (*1*,*5*,*26*). Samples coming from sentinel sites in areas of Haiti subjected to future drug pressure will be of particular focus for timely acquisition of molecular drug-resistance data.

Investigations of *P. falciparum* resistance to chloroquine in Haiti have been numerous and ongoing since 1981. Even the first reports in the early 1980s found in vivo and in vitro evidence for possible resistance to chloroquine (*27*,*28*), which had been in use in Haiti at least since the 1950s (*29*). With the advent of mutations in the *Pfcrt* gene serving as molecular markers for chloroquine resistance (*18*,*19*), the *Pfcrt* K76T haplotype has been reported sporadically in Haiti since 2009, but always at very low rates (*2*,*30*). Our study found that all 741 isolates collected from sites around Haiti that were successfully sequenced for *Pfcrt* have codons 72–76 of the CVMNK genotype, which indicate chloroquine susceptibility (*31*,*32*). Our data and previous reports indicate that chloroquine-resistant *Pfcrt* alleles evidently are rarely found in Haiti, a finding which supports the continued use of this drug for the primary treatment of malaria in Haiti. As antimalarial treatment practices are improved nationwide during elimination efforts, ongoing monitoring will be necessary to ensure the appropriate use of this important drug in the country.

During malaria elimination efforts in Haiti in the 1960s, MDA campaigns distributed chloroquine/pyrimethamine tablets (at an adult dose of 600 mg chloroquine and 50 mg pyrimethamine) to the population in areas with a high level of transmission, with particular focus on the southern peninsula (*33*). Drug uptake in the population was high (>90%), and in total, >2 million persons in Haiti were reached by these campaigns, some receiving up to 15 rounds of MDA. The MDA campaigns were begun in 1964, and by 1966, widespread success in reducing parasite rates led to focal drug distribution in outbreak areas and eventual cessation in the late 1960s (*34*). With termination of malaria elimination efforts in the 1970s, *P. falciparum* cases rapidly increased throughout the nation to the point where most of the population was again at risk for exposure (*34*).

One of the most consistent findings in this 2016–2017 study was the pervasive prevalence of the *Pfdhfr* S108N mutation in Haiti; 47% of all successfully sequenced isolates showed this polymorphism. This point mutation is well known to develop under pyrimethamine drug pressure (*18*), and previous studies in Haiti have found evidence to both in vivo and in vitro pyrimethamine resistance (*35*,*36*), and, more recently, the specific S108N mutation at frequencies of 36% (*2*) and 33% (*37*). Because the *P. falciparum* parasite population in Haiti has been shown to be of low genetic diversity and distinct from that of South America (*2*,*3*,*38*), presence of this genotype in Haiti probably could be traced back to MDA campaigns in the 1960s. It is remarkable that *P. falciparum* parasites in Haiti would continue to carry this polymorphism after 50 years, although the S108N mutation has been shown to be the first to arise during pyrimethamine drug pressure (*39*,*40*). This observation is consistent with the hypothesis that the S108N mutation does not affect fitness of the parasites carrying this allele (*41*). S108N mutation confers only low-level resistance to pyrimethamine, and the presence of >3 mutations (leading to the N51I/C59R/S108N triple mutant) is required for high-level resistance (*42*–*45*). Outside of pyrimethamine, the atovaquone/proguanil chemoprophylactic regimen often used by travelers to Haiti might also have the potential to induce drug pressure on the *Pfdhfr* gene and might lose efficacy if multiple codon mutations would arise in the gene (*46*). Because the *P. falciparum* parasite population in Haiti is currently only showing incomplete penetrance of the S108N mutation, if SP were used for MDA in Haiti, then this ongoing drug-resistance surveillance program will be crucial to detect any emergence of *Pfdhfr* double or triple mutants, especially in conjunction with the A437G and K540E mutations in the *Pfdhps* gene (*47*). The presence of 1 *Pfdhfr* triple mutant in Cazale and 1 *Pfdhps* single mutant in Les Anglais is notable, but both represent well under 1% of the parasite isolates in the our study, which is consistent with previous reports (*2*,*37*).

Categorizing the samples by quantified parasite DNA levels was very predictive of success in Sanger sequencing for the *Pfdhfr* and *Pfdhps* genes, although *Pfcrt* Sanger sequencing performed well at any level of DNA content. Using this strategy of DNA quantification will assist in excluding samples for *Pfdhfr* and *Pfdhps* Sanger sequencing that would have a low likelihood of interpretable results. Although the success rate for Sanger sequencing of *Pfdhfr* and *Pfdhps* was low compared with the success rate for *Pfcrt*, especially in low-density parasitemia samples, resistant chloroquine or highly resistant SP resistant genotypes clearly are very rare across Haiti. Furthermore, our results support the rationale for using SP for MDA in certain areas of Haiti with the aim of interrupting *P. falciparum* transmission as part of malaria elimination efforts. If different antimalarial drug strategies are proposed for Haiti in the future, this nationwide sentinel surveillance system could also be used for monitoring of other putative genetic markers of resistance. In addition, employing next-generation sequencing methods will also enable the comprehensive characterization of this parasite population for well-established resistance markers in other target genes besides only *Pfcrt, Pfdhfr,* and *Pfdhps*. Continued monitoring for drug-resistance markers, especially during MDA, will be critical for detecting any potential emergence of highly resistant genotypes associated with resistance to SP and chloroquine.

Appendix Additional information about nationwide monitoring for *Plasmodium falciparum* drug-resistance alleles to chloroquine, sulfadoxine, and pyrimethamine, Haiti, 2016–2017.
