# Supplementary material for: Nationwide Monitoring for Plasmodium falciparum Drug-Resistance Alleles to Chloroquine, Sulfadoxine, and Pyrimethamine, Haiti, 2016–2017
Source: Emerg Infect Dis. 2020 May;26(5):902–9. doi: 10.3201/eid2605.190556 (PMC7181918; doi:10.3201/eid2605.190556)
Supplement: Appendix — Additional information about nationwide monitoring for Plasmodium falciparum drug-resistance alleles to chloroquine, sulfadoxine, and pyrimethamine, Haiti, 2016–2017. [file 19-0556-Techapp-s1.pdf]

Article DOI: https://doi.org/10.3201/eid2605.190556

Nationwide Monitoring for *Plasmodium falciparum* Drug-Resistance Alleles to Chloroquine, Sulfadoxine, and Pyrimethamine, Haiti, 2016–2017

Appendix

Appendix Table 1. Primers and PCR conditions for *Pfcrt*, *Pfdhfr*, and *Pfdhps* amplification*

Gene target	Primer (5′→3′)	Thermocycling conditions†
Pfcrt		
Primary rxn		94°/5 min; [95°/30 sec; 56°/30 sec; 65°/1 min] ×30 ; 65°/5 min
Forward	CATTGTCTTCCACATATATGACATAAA	
Reverse	TTGGTAGGTGGAATAGATTCTCTT	
secondary rxn		94°/5 min; [95°/30 sec; 60°/30 sec; 65°/1 min] ×30 ; 65°/5 min
Forward	CCGTTAATAATAAATACACGCAG	
Reverse	AATTTCCCTTTTTATTTCCAAATAAGG	
Sequencing rxn	AATTTCCCTTTTTATTTCCAAATAAGG	96°/1 min; [96°/10 sec; 50°/5 sec; 60°/4 min] ×25
Pfdhfr		
Primary rxn		95°/5 min; [95°/30 sec; 50°/30 sec; 68°/1 min] ×30; 68°/5 min
Forward	TCCTTTTTATGATGGAACAAG	
Reverse	AGTATATACATCGCTAACAGA	
secondary rxn		95°/5 min; [95°/30 sec; 52°/30 sec; 68°/1 min] ×30; 68°/5 min
Forward	TTTATGATGGAACAAGTCTGC	
Reverse	ACTCATTTTCATTTATTTCTGG	
Sequencing rxn	TTTATGATGGAACAAGTCTGC	96°/1 min; [96°/10 sec; 50°/5 sec; 60°/4 min] ×25
Pfdhps		
PCR rxn		95°/5 min; [95°/30 sec; 53°/30 sec; 68°/1 min] ×40; 68°/5 min
Forward	AACCTAAACGTGCTGTTCAA	
Reverse	TCATTTTGTTGTTCATCATGT	
Sequencing rxn		96°/1 min; [96°/10 sec; 50°/5 sec; 60°/4 min] ×25
Forward	CTATAGTGTAGTTCTAATGC	
Reverse	CAACTAAATCATTATCAACACATTC	

*rxn, reaction.
†Initialization; [denaturation; annealing; extension] x cycles; final elongation

Appendix Table 2. Number of samples successfully sequenced (and as percentage of total samples selected from that site) for reporting of putative drug resistance codons for genes *Pfcrt*, *Pfdhfr*, *Pfdhps*, by participating healthcare facility, Haiti 2016–2017*

	Pfcrt				*Pfdhfr*					*Pfdhps*				
Site (no. samples)	C72S	M74I	N75E	K76T	C50R	N51I	C59R	S108N	I164L	S436X†	A437G	K540E	A581G	A613X‡
Arnaud (211)	208 (99)	208 (99)	208 (99)	208 (99)	135 (64)	135 (64)	135 (64)	135 (64)	135 (64)	155 (73)	155 (73)	129 (61)	141 (67)	135 (64)
Capotille (13)	11 (92)	11 (92)	11 (92)	11 (92)	9 (75)	9 (75)	9 (75)	9 (75)	9 (75)	6 (50)	6 (50)	6 (50)	5 (42)	4 (33)
Cazale (23)	23 (100)	23 (100)	23 (100)	23 (100)	16 (70)	16 (70)	16 (70)	15 (65)	15 (65)	8 (35)	8 (35)	7 (30)	7 (30)	8 (35)
Dalphane (21)	21 (100)	21 (100)	21 (100)	21 (100)	19 (90)	19 (90)	19 (90)	18 (86)	19 (90)	0 (0)	0 (0)	0 (0)	0 (0)	0 (0)
Lavanneau (12)	12 (100)	12 (100)	12 (100)	12 (100)	4 (33)	4 (33)	4 (33)	6 (50)	4 (33)	1 (8)	1 (8)	1 (8)	1 (8)	1 (8)
Les Anglais (211)	195 (92)	195 (92)	195 (92)	195 (92)	152 (72)	152 (72)	152 (72)	152 (72)	152 (72)	138 (65)	138 (65)	123 (58)	141 (67)	140 (66)
Pignon (2)	2 (100)	2 (100)	2 (100)	2 (100)	2 (100)	2 (100)	2 (100)	2 (100)	2 (100)	0 (0)	0 (0)	0 (0)	0 (0)	0 (0)
Roseaux (68)	62 (94)	62 (94)	62 (94)	62 (94)	48 (71)	48 (71)	48 (71)	47 (69)	48 (71)	39 (57)	39 (57)	24 (35)	34 (50)	32 (47)
Sainte-Hélène (199)	190 (95)	190 (95)	190 (95)	190 (95)	150 (75)	150 (75)	150 (75)	149 (75)	150 (75)	138 (69)	138 (69)	116 (58)	136 (68)	138 (69)
Thomonde (18)	17 (94)	17 (94)	17 (94)	17 (94)	16 (89)	16 (89)	16 (89)	15 (83)	15 (83)	3 (17)	3 (17)	0 (0)	0 (0)	0 (0)
Total (778)	741 (95)	741 (95)	741 (95)	741 (95)	551 (71)	551 (71)	551 (71)	548 (70)	549 (71)	488 (63)	488 (63)	406 (52)	465 (60)	458 (69)

*Values are no. (%) unless indicated.
†S436A, S436Y, or S436F mutations.
‡A613S or A613T mutations.

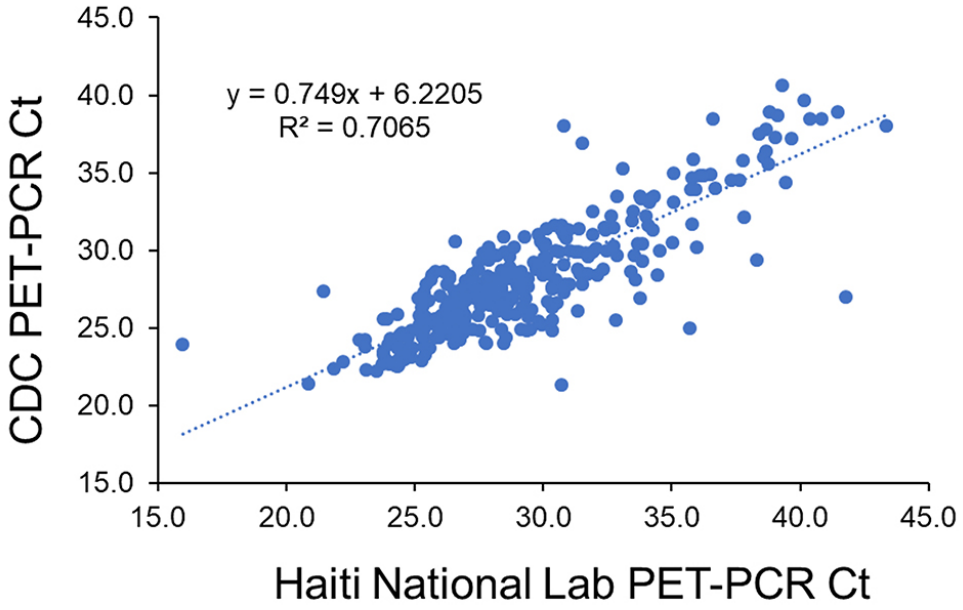

Appendix Figure. Concordance of PET-PCR cycle threshold (C_t) values from first 325 samples in drug resistance survey, Haiti 2016. Scatterplot of C_t values for PET-PCR reactions as run at the Haitian National Laboratory versus the same person's sample with DNA extraction and PET-PCR assay at CDC in Atlanta, Georgia.
